# Correlation Analysis between GlpQ-Regulated Degradation of Wall Teichoic Acid and Biofilm Formation Triggered by Lactobionic Acid in *Staphylococcus aureus*

**DOI:** 10.3390/foods11213438

**Published:** 2022-10-29

**Authors:** Wanwan Hou, Shimo Kang, Jiang Chang, Xiaorong Tian, Chunlei Shi

**Affiliations:** MOST-USDA Joint Research Center for Food Safety, School of Agriculture and Biology, and State Key Laboratory of Microbial Metabolism, Shanghai Jiao Tong University, Shanghai 200240, China

**Keywords:** *Staphylococcus aureus*, wall teichoic acid, GlpQ, biofilm formation

## Abstract

*Staphylococcus aureus* biofilms are a serious problem in the food industry. Wall teichoic acid (WTA) is crucial in *S. aureus* biofilm formation. Overexpression of the WTA-hydrolyzing enzyme glycerophosphoryl diester phosphodiesterase (GlpQ), induced by lactobionic acid (LBA), may be related to biofilm formation. We investigated the relationship between the regulation on GlpQ degradation of WTA by LBA and *S. aureus* biofilm formation. LBA minimum inhibitory concentration for *S. aureus* was 12.5 mg/mL. Crystal violet staining revealed the LBA-mediated inhibition of *S. aureus* adhesion and biofilm formation. RT-qPCR revealed the repressed expression of adhesion-related genes by LBA. Scanning electron microscopy revealed the obvious disruption of *S. aureus* surface structure, confirming the repression of *S. aureus* adhesion and biofilm formation by LBA. Native-PAGE results suggested that the WTA content of *S. aureus* was reduced under the inhibition of LBA. Additionally, LBA induced the overexpression of *glpQ*. Combined with our previous work, these results suggest that *glpQ* is induced in *S. aureus* to function in WTA degradation with the addition of LBA, resulting in decreased WTA content and subsequent reduction of adhesion and biofilm formation. The findings provide new insight into the degradation mechanism of *S. aureus* WTA and indicate the potential of LBA as an anti-biofilm agent.

## 1. Introduction

*Staphylococcus aureus* is a common foodborne pathogen that can easily adhere to and form biofilms on various food processing equipment surfaces, such as those in dairy and meat processing. Within biofilms, the bacteria can remain viable even after cleaning and disinfecting treatment. This can lead to cross contamination of foods that greatly increase the risk of foodborne disease outbreaks [1,2]. In addition, *S. aureus* biofilms facilitate the spread of antibiotic resistance by promoting horizontal gene transfer [3]. Therefore, the biofilm formation of *S. aureus* is considered one of the most common causes of food contamination and clinical infections nowadays.

Wall teichoic acids (WTAs) are phosphate-rich, anionic-charged glycopolymers found on the cell wall of many Gram-positive bacteria, comprising approximately 60% of the dry weight of cell walls. WTAs play pivotal roles in *S. aureus* adhesion to the surface and initiation of biofilm formation [4,5]. WTAs are composed of a backbone containing up to 40 glycerol–3–phosphate (GroP) or ribitol–5–phosphate (RboP) molecules [6]. Most *S. aureus* variants synthesize RboP-WTA (e.g., *S. aureus* N315). The GroP or RboP backbone is covalently attached to the cell wall peptidoglycan (PNG) via a conserved linkage unit. In *S. aureus*, the unit includes two GroP units followed by *N*-acetylmannosamine and *N*-acetylglucosamine linked to the *N*-acetylmuramic acid via a phosphodiester bond [7]. The WTA chains are often modified with glycosyl and D-alanine residues at different locations [8]. These modifications are considered to be key determinants of *S. aureus* biofilm formation due to the resulting change of the surface charge [9]. The absence of *S. aureus* WTAs affects biofilm formation and cell aggregative behavior of *S. aureus* [5,9]. Thus, WTA synthesis and degradation is a promising target for prevention of *S. aureus* adhesion and biofilm formation. 

Some teichoicases or WTA-hydrolyzing enzymes have been identified in Gram-positive bacteria and bacteriophages. These enzymes catalyze the degradation of chain of WTAs by acting on phosphodiester bonds in the WTA backbone [10]. These enzymes include GP12, φ29, GlpQ, and PhoD [11,12]. The glycerophosphodiesterase GlpQ is secreted by *S. aureus* as an exolytic WTA-hydrolyzing enzyme with strict stereospecificity. GlpQ sequentially cleaves off GroP entities from the exposed end of WTA [12]. The expression of GlpQ is usually upregulated during phosphate limitation as a compensatory mechanism to maintain the survival of bacteria by using the cleaved free GroP as the sole source of phosphate [11]. GlpQs involved in the WTA degradation pathway have been explored in GroP-WTA type G^+^ bacteria, including *Bacillus subtilis* and coagulase-negative staphylococci. Little is known about RboP-WTA type G^+^ bacteria. 

Lactobionic acid (LBA) is an organic acid naturally found in the Caspian Sea yogurt and is most known for its antimicrobial, antioxidant, chelating, acidulant, moisturizing properties [10]. We previously described the significant upregulation of the protein level of WTA-hydrolyzing enzyme GlpQ in *S. aureus* N315 after inducement with LBA. Furthermore, the proteins involved in adhesion and biofilm formation were shown to be significantly downregulated under the inhibition of LBA [13]. These findings suggest that GlpQ overexpression may be related to *S. aureus* biofilm formation. However, whether GlpQ can degrade RboP-WTA type G^+^ bacteria is unknown. The highly conserved WTA linkage unit in different bacterial species contains two GroP units. It is also unknown whether GlpQ can degrade RboP-WTA by cleaving the phosphodiester bond connecting with the two GroP units.

The present study explored the possible correlation between WTA-hydrolyzing enzyme GlpQ and *S. aureus* biofilm formation. The findings, which demonstrate such a correlation, provide a new perspective that will help clarify the degradation mechanism of WTA. The findings also provide a new method for the prevention and control of *S. aureus* biofilm formation.

## 2. Materials and Methods

### 2.1. Reagents, Bacterial Strains, and Growth Conditions

The N315 and SJTUF21564 *S. aureus* strains used in this study were stored in our laboratory at −80 °C until used. N315 is a methicillin-resistant strain of *S. aureus* (MRSA), which was isolated in 1982 from the pharyngeal smear of a Japanese patient [14]. SJTUF21564 is a strain of *S. aureus* with strong biofilm formation ability that was isolated from chicken meat in our laboratory in 2018. Strong biofilm forming ability of SJTUF21564 was indicated by the optical density at 595 nm (OD_595_) that is over four-times greater than the OD of blank (ODc) at 595 nm. In the present study, weak biofilm production was defined as ODc < OD < 2 × ODc. Heavy biofilm production was defined as OD > 4 × ODc [15]. All strains were cultured in tryptic soy broth (TSB) medium (Land Bridge, Beijing, China) and incubated at 37 °C. Lactobionic acid (LBA; ≥98% purity) was purchased from BIDE (Shanghai, China; BD135770).

### 2.2. Minimum Inhibitory Concentration (MIC)

In vitro susceptibility (MIC determination) was performed using the CLSI broth microplate assay guidelines [16]. In brief, LBA was primarily dissolved in dimethyl sulfoxide (DMSO, <1%, *v*/*v*) and was subsequently diluted in sterilized distilled water. Serial two-fold dilutions of LBA at concentrations ranging from 512 to 2 mg/mL were established in 96-well plates containing 100 µL of TSB broth. All wells were inoculated with 2 µL bacterial cells at an OD_600_ = 0.50 and incubated at 37 °C for 24 h. The minimal concentration of LBA which inhibited visible growth of bacteria represented the MIC. All assessments were repeated in triplicate.

### 2.3. Biofilm Formation Assay

The microtiter plate biofilm formation assay is a method for the study of early biofilm formation on abiotic surfaces [17]. Stationary phase *S. aureus* cells were resuspended in physiological NaCl solution to an OD_600_ of 0.80 and diluted 1:100 in TSB broth. Wells of a 96-well plate were inoculated with 200 μL of this suspension, and LBA was added to the final concentrations, which were 1 × MIC and 0.5 × MIC, then incubated for 24 h at 37 °C. Each well was rinsed twice to remove planktonic cells. The adherent biofilms were stained with crystal violet (0.1% in distilled water) for 15 min and washed with PBS three times. After visual observation, the adhering dye was dissolved in 100% alcohol. The determined optical density at 595 nm was used to quantify the biomass. The group without LBA was the control.

### 2.4. Light Microscopy of S. aureus Adhesion

*S. aureus* was incubated in TSB broth overnight and diluted 200-fold. An amount of 300 μL diluted suspensions and 30 mL TSB broth were added to each well of a 6-well plate. LBA was then added to the final concentrations, which were 1 × MIC and 0.5 × MIC. Coverslips were incubated in wells of 6-well plates for 24 h at 37 °C. The coverslips were removed and rinsed with PBS to remove the suspended bacteria on the surface, dried at room temperature, stained with 1% crystal violet for 15 min, and washed three times with sterile water to remove the floating color. Light microscopy (400×) was used to observe the biofilms [18]. The group without LBA as the control.

### 2.5. Scanning Electron Microscopy (SEM) of S. aureus Biofilms

*S. aureus* in TSB broth was added into wells of a 6-well tissue culture plate. LBA was then added to the final concentrations which were 1 × MIC and 0.5 × MIC. Sterile glass slides (10mm × 10 mm) were added to each well and incubated at 37 °C for 24 h. Each cover glass covered by biofilm was washed twice with PBS to remove non-adherent bacteria, and examined by field emission-SEM (FE-SEM) using a Sirion 200 microscope (FEI, Hillsboro, OR, USA) as previously described with minor modifications [19]. The biofilms were fixed in 1 mL of 2.5% glutaraldehyde for 12 h at 4 °C. The fixed cells were dehydrated in a gradient of 30, 50, 70, 90, 95, and 100% ethanol (*v*/*v*). After freeze-drying and gold coating, the *S. aureus* biofilm samples were observed by high-resolution FE-SEM using a Sirion 200 microscope at an accelerating voltage of 20 kV and 20,000× magnification. The group without LBA as the control.

### 2.6. Determination of WTA by Native-Polyacrylamide gel Electrophoresis (Native-PAGE)

#### 2.6.1. Extraction of WTA

WTA was extracted with diluted NaOH as described previously with minor modifications [20]. Briefly, an overnight (>16 h) culture of *S. aureus* cells (0.3 mL) was subcultured into 30 mL fresh TSB broth. After the 14 h incubation, LBA (1 × MIC, 0.5 × MIC) was added and treated for 2 h. After further cultivation at 37°C with 220 rpm shaking, cells were harvested by centrifugation (4000× *g* and 4 °C for 20 min) when the OD_600_ reached 0.6. WTA was isolated as described previously [19]. In brief, the cell pellet was washed with 50 mM MES (pH 6.5) and resuspended in 4% sodium dodecyl sulfate (SDS) in 50 mM MES (pH 6.5). The cell suspension was boiled at 95 °C for 1 h, pelleted at 14,000× *g* for 10 min, and washed once with 2% (*w*/*v*) NaCl in 50 mM MES (pH 6.5) and five times with 50 mM Tris-HCl (pH 8). Cell sacculi were further digested with 2 mL of 0.1 mg/mL proteinase K in the presence of 20 mM Tris-HCl (pH 8.0) and 0.5% (*w*/*v*) SDS) at 50 °C for 4 h, washed three times with deionized water, and incubated in 1 mL of 0.1 M NaOH at room temperature and 120 rpm for 16 h. The dissolved WTA solution was collected through centrifugation at 14,000× *g* for 15 min and stored at 4 °C for subsequent SDS-PAGE analysis.

#### 2.6.2. Native-PAGE Analysis of WTA

The collected WTA samples were electrophoresed at 4 °C in a Protean II electrophoresis cell (Bio-Rad, Hercules, CA, USA) for 18 h using a current of 15 mA/gel. The running buffer contained 0.1 M Tris base and 0.1 M Tricine at pH 8.2. The gel was developed using the Alcian blue/silver staining method as described previously [21].

### 2.7. RNA Isolation and qRT-PCR

An aliquot (200 μL) of overnight pure cultures of both *S. aureus* strains was individually added to 20 mL TSB broth, and incubated at 37 °C with shaking at 220 rpm until log or stationary growth phases. The total RNA of all *S. aureus* strains was extracted with SPARKeasy Improved Bacteria RNA kit (AC0402; SparkJade, Jinan, China). Three biological replicates were prepared for each strain. RNA was demonstrated to be intact by 1% agarose gel electrophoresis; its concentration was quantified using a NanoDrop 2000 spectrophotometer (Thermo Fisher Scientific, Waltham, MA, USA).

The total RNA of the strains was reverse-transcribed to generate cDNA with a SPARK script II RT Plus Kit (AG0304; SparkJade) for real-time quantitative PCR. Amplification of samples was performed using the 2 × SYBR Green qPCR Mix (With ROX; AH0104; SparkJade) in an Eppendorf Mastercycler ep realplex 4 system (Eppendorf, Mannheim, Germany). The samples were tested in triplicate in three independent experiments using the following conditions: 94 °C for 3 min followed by 40 cycles of amplification at 94 °C for 15 s, 55 °C for 15 s, and 72 °C for 25 s. The relative transcriptional levels of the chosen genes were calculated using the 2^ΔΔCT^ threshold cycle (C_T_) method [13]. The expression of *rpoB* was determined as normalization control. The genes used in this study are listed in Table 1.

### 2.8. Statistical Analysis

Standard deviations, average, analysis of variance, *t*-test, graphs, and other statistical analyses were performed using GraphPad prism 8.0.1 software (GraphPad Software, San Diego, CA, USA). A *p*-value < 0.05 or <0.01 indicated statistical differences between means.

## 3. Results and Discussion

### 3.1. In Vitro Antibacterial Susceptibility Testing

In vitro antibacterial susceptibility testing was detected by broth micro-dilution method to determine MICs of LBA on *S. aureus* strains N315 and SJTUF21564 to evaluate the effects of LBA on both strains. The MICs of LBA against N315 and SJTUF21564 were both 12.5 mg/mL. It is worth noting that the MIC values that we measured differed from our previous study; the prior MIC value of LBA for N315 was 18.75 mg/mL [13]. The decreased MIC observed presently could be attributed to the change of purity of LBA obtained from different manufacturers. Antibiotics are often present at the site of infection at sub-inhibitory concentrations (sub-MICs) that damage but do not kill bacteria. Sub-MICs of various antibacterials can modify the molecular architecture of the external surface of bacteria and some bacterial functions [22]. Furthermore, antibacterial agents such as epigallocatechin gallate and shikimic acid, at sub-MIC concentrations, can significantly reduce the biofilm biomass [23,24]. The foregoing observations indicate that sub-MIC concentrations are more suitable for the antibiofilm assays. Hence, the sub-MIC concentration of LBA at 6.25 mg/mL (0.5 × MIC) was also chosen for the following biofilm formation assays.

### 3.2. Effect of LBA on Biofilm Formation of S. aureus

**Figure 1:**
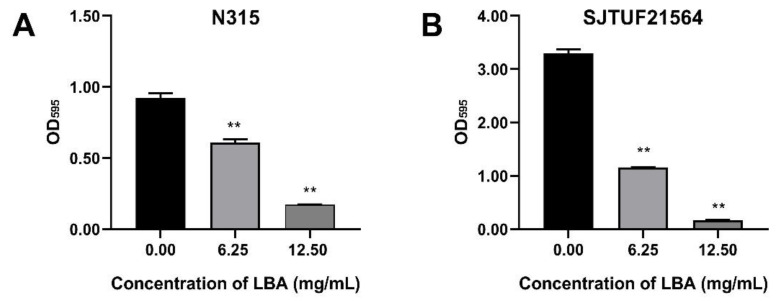
Effect of LBA on biofilm formation of *S. aureus* strain N315 (**A**) and SJTUF21564 (**B**). The values on the X-axes correspond to the following: 0.00, untreated control; 6.25, 0.5 × MIC; and 12.50, 1 × MIC. **, statistical significance at *p* < 0.01 by analysis of variance.

*S. aureus* strains N315 and SJTUF21564 were tested to determine their biofilm-forming ability by crystal violet staining assay. SJTUF21564 was superior in forming biofilms to N315 and to all *S. aureus* strains maintained in our laboratory. As shown in Figure 1, the OD_595_ value in untreated SJTUF21564 (3.30) was more than three-times higher than untreated N315 (0.92), and the OD_595_ values both in untreated SJTUF21564 (24.61 fold) and untreated N315 (6.88 fold) were over four-times greater than the ODc at 595 nm, indicating that both SJTUF21564 and N315 have heavy biofilm-producing abilities and the ability of SJTU21564 was obviously higher than N315.

Crystal violet staining assay revealed that LBA at the concentrations of 0.5 × MIC and 1 × MIC significantly (*p* < 0.01) inhibited the biofilm formation of N315 with a respective decrease of 33.9% and 81.4% (Figure 1A) and the biofilm formation of SJTUF21564 by 65.4% and 95.1%, respectively (Figure 1B). The biofilm formation ability of SJTUF21564 was almost three times greater than that of N315, and LBA was more effective in inhibiting SJTUF21564. The role of organic acid on biofilm inhibition of *S. aureus* evident in the crystal violet staining assay has been previously reported in various ways, such as shikimic acid [24], boswellic acid [25], and citric acid [26]. In the present study, another organic acid LBA also significantly inhibited the formation of *S. aureus* biofilms (*p* < 0.01).

### 3.3. Effect of LBA on Adherence of S. aureus

**Figure 2:**
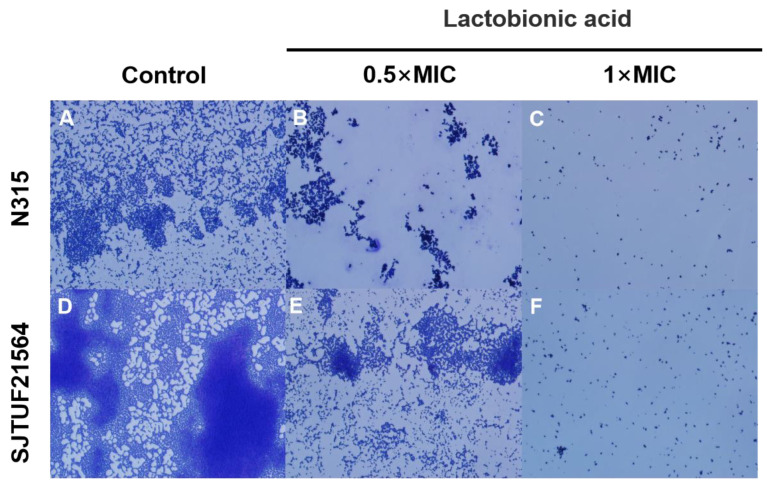
Light microscopy images of *S. aureus* biofilms inhibited with LBA at different concentrations. (**A**,**D**) are the controls, (**B**,**E**) are induced with 0.5 × MIC, and (**C**,**F**) are induced with 1 × MIC.

The adhesion of *S. aureus* after crystal violet staining was observed by light microscopy. After 24 h incubation, larger and uniform bacterial clusters adhered to the surface of slides in untreated groups (Figure 2). The biofilms formed by SJTUF21564 were tighter; some individual colonies were observed locally on the slides, and some colonies merged into small clusters (Figure 2A). In contrast, biofilms formed by N315 were in less density (Figure 2D).

However, small irregularly shaped biofilms could still be observed in SJTUF21564, with even fewer clusters observed from biofilms in N315 (Figure 2B,E). When the concentration of LBA was increased to 1 × MIC, both N315 and SJTUF21564 were no longer evident as patches of biofilms (Figure 2C,F). These results indicate that LBA could potently inhibit *S. aureus* adhesion and biofilm formation. Therefore, LBA may be useful as an antibiofilm agent and may play an important role in adhesion and biofilm formation of *S. aureus*. The adherence of *S. aureus* to host cells is the first step in colonization and infection [27]. An important factor in the initial interaction between *S. aureus* and its host is the ability of the bacterium to adhere to the host cell surface [28,29]. Accordingly, we investigated the expression of adhesion-related genes of *S. aureus*.

**Figure 3:**
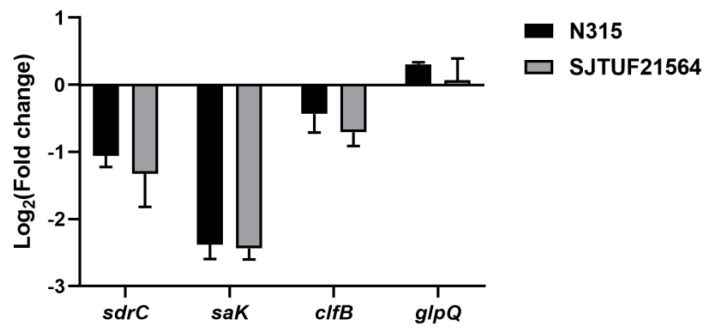
Expression of adhesion genes and *glpQ* in *S. aureus* after treatment with 0.5 × MIC LBA by RT−qPCR.

Inhibition with 0.5 × MIC LBA significantly decreased the expression of adhesion-associated genes *sdrC* and *saK* in *S. aureus* strains N315 and SJTUF21564 (*p* < 0.01; Figure 3). In addition, the adhesion-associated gene *clfB* also displayed a trend of downregulation in both strains; the downregulation was not significant in N315 (Figure 3). The results indicate that LBA could inhibit the adhesion of multidrug resistant and avid biofilm forming *S. aureus*.

Serine–aspartate repeat C (*sdrC*) is a differential adhesion gene associated with *S. aureus*. The gene was screened in our previous work. Presently, *sdrC* was significantly downregulated, in agreement with our previous proteomic results (*p* < 0.05) [13]. The *sdr* family of proteins have a primary role in the development of *ica*-independent biofilms. SdrC and fibronectin/fibrinogen-binding proteins have been implicated in biofilm matrix formation [30,31]. SdrC forms low-affinity homophilic bonds that promote cell–cell adhesion, as well as mediating strong cellular interactions with hydrophobic surfaces during initial attachment of bacteria and early development of biofilms [31]. Previous studies also demonstrated the contribution of *sdrC* and *sdrD* to bacterial attachment to human nasal epithelial cells and medical equipment [32,33].

The second gene *saK* encodes staphylokinase, a potent immune modulatory factor of *S. aureus*. The SAK protein is significantly induced in community-associated *S. aureus* and inhibits biofilm formation in a plasminogen-dependent manner (*p* < 0.05) [34]. The expression of *saK* may be beneficial for the more avid formation of biofilms, cell invasion, and evasion of host immunity [35,36].

The third gene *clfB* is a common gene related with biofilm formation in *S. aureus*. *clfB* gene expression increases during the growth of biofilms [37]. Abraham et al. reported that *clfB* can mediate biofilm formation. In the condition of Ca^2+^ ion exhaustion, when the *clfB* gene is knocked out, biofilm growth can be significantly inhibited (*p* < 0.05) [38]. The ClfB protein can also bind to the fibrinogen, activating it to form fibronectin and promoting agglutination, an essential process in the second stage of biofilm formation [39].

The downregulation of these genes after inhibition with LBA could be the potential reason for this drug’s inhibitory effect against *S. aureus* biofilm formation. This strategy may be beneficial in the treatment of chronic *S. aureus* infections and as a means of reducing its transmission capabilities in clinical settings.

### 3.4. SEM Visualization of S. aureus Biofilm Formation

**Figure 4:**
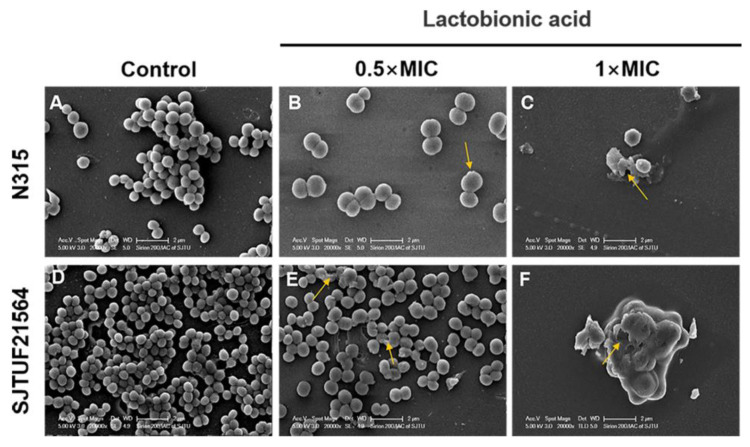
SEM images of biofilm formation of *S. aureus* inhibited with LBA at different concentrations. (**A**,**D**) are the controls, (**B**,**E**) are induced with 0.5 × MIC, and (**C**,**F**) are induced with 1 × MIC. Yellow arrows show disrupted cells.

The adherence analysis revealed that LBA could inhibit the biofilm formation in the early stage by affecting the initial adherence of *S. aureus* cells. To better understand the process by which LBA inhibits biofilm formation of *S. aureus*, SEM observations of *S. aureus* biofilms formed in the absence and presence of LBA were performed. SEM images (20,000× magnification) of *S. aureus* biofilms inhibited with LBA clearly showed the dose-dependent changes of the biofilm formation and architecture of *S. aureus* (Figure 4). In the untreated controls, the majority of the bacteria in the biofilms were fully spherical with no deformation. The bacteria were closely connected with each other and formed a dense three-dimensional network on the cover glass. Bacteria within biofilms displayed a smooth surface and spherical contour (Figure 4A,D). Consistent with the results of semi-quantitative adhesion observed by light microscopy, the bacterial biofilms formed by SJTUF21564 were more compact (Figure 2B,F). After inducement with 0.5 × MIC LBA, the biofilms displayed fewer cells that were loosely attached to the glass slides; parts of the cell surfaces were damaged (Figure 4B,E). Furthermore, depressions appeared on the surface of most SJTUF21564 bacteria and a minority were disrupted. After inducement with 1 × MIC, the two strains were unable to form biofilms and the cells were severely broken (Figure 4C,F). Additionally, LBA-treated cells enlarged in size after LBA treatment compared to the untreated group, maybe due to the LBA-treated cells being unable to withstand the osmotic pressure in liquid medium [40]. The SEM observations showed that LBA was able to reduce *S. aureus* adhesion and inhibit biofilm formation by disrupting the surface structure of *S. aureus* cells.

These findings were also consistent with our previous observation that LBA has significant antibacterial effects, and disrupts the integrity of cell walls and cell membranes, as well as the content and activity of bacterial proteins [14]. It can bind to genomic DNA, rendering the cells incapable of normal viability [14]. Similarly, LBA can also inhibit biofilm of other strains. Fan et al. found that LBA inhibited biofilm formation of *Salmonella* Typhimurium [41] and *Vibrio parahaemolyticus* [42]. Another study demonstrated that LBA was able to damage the cell wall and cell membrane of *S. aureus*, resulting in the change of the ultrastructure and membrane dysfunction, up to cytoplasmic (electrolytes, proteins, and nucleic acids) leakage. LBA exerts its antibacterial activity by breaking down the structure of the bacterial cell wall and membrane. This damage may be the main mechanism by which LBA inhibits and kills *S. aureus* [43,44].

### 3.5. Effect of LBA on WTA Content

WTA is an important factor involved in cell adhesion and plays a key role in the first step of biofilm formation [9]. Accordingly, we analyzed the extracted WTA by native-PAGE with silver staining to provide a semi-quantitative measure of total polymer [45] to characterize the effect of LBA on WTA content in *S. aureus*.

**Figure 5:**
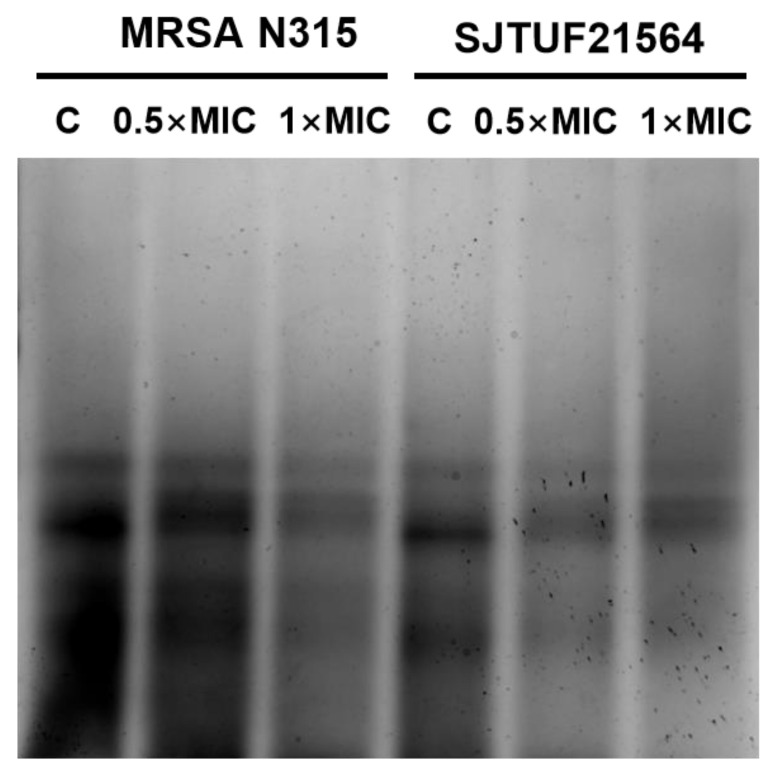
WTA-PAGE analysis of *S. aureus* after inhibition with LBA at different concentrations. Samples shown here were resolved in polyacrylamide gels and visualized with Alcian blue/silver staining.

From Figure 5, it can be seen that WTA extracted from untreated *S. aureus* appeared as discrete size polymers (Figure 5). *S. aureus* treated with 0.5 × MIC LBA and 1 × MIC LBA displayed a decrease in WTA polymer levels in a dose-dependent manner. The findings indicate that the WTA content of *S. aureus* was reduced in a dose-dependent manner after LBA treatment (as observed by reduced staining of polymers levels). This trend was consistent with the results of the reduction in adhesion and the amount of biofilm formation. Adhesion, biofilm formation, and the WTA content of both *S. aureus* strains were reduced in a dose-dependent manner after LBA exposure. The presence of WTA in *S. aureus* affects the biofilm forming ability and cell aggregation behavior, which eventually leads to reduced biofilm formation [5]. The collective findings support the speculation that decreased adhesion and suppressed biofilm formation by *S. aureus* exposed to LBA may be related to the reduction in WTA content.

### 3.6. Analysis of WTA Hydrolase GlpQ Associated with S. aureus Biofilm Formation

In the present study, we determined the gene expression of WTA-hydrolyzing enzyme GlpQ secreted by *S. aureus* using RT-qPCR. The results revealed upregulated *glpQ* expressions in both *S. aureus* strains after exposure to 0.5 × MIC LBA (*p* < 0.05) [13]. In a previous study, the protein expression of GlpQ were assessed by untargeted and targeted quantitative proteomics. The analyses revealed significantly upregulated GlpQ expression after exposure to 0.5 × MIC LBA (*p* < 0.05, Figure 3) [13]. It can be seen that the trend of protein expression of GlpQ was consistent with its gene expression. In addition, the degree of early stage adhesion and aggregation of both *S. aureus* strains were reduced, and the amount of biofilm formation was significantly inhibited. These findings suggest that the upregulation of *glpQ* expression and GlpQ production is related to *S. aureus* biofilm formation.

GlpQ is the first reported WTA-hydrolyzing enzyme from *S. aureus*. It is usually significantly upregulated under phosphate-restricted conditions only in GroP-WTA type *S. aureus* (*p* < 0.05), allowing the bacteria to live normally [11]. Studies on the roles of GlpQ in the WTA degradation pathway have involved GroP-WTA type G^+^ bacteria, such as *Bacillus subtilis* and coagulase-negative staphylococci. In GroP-WTA type G^+^ bacteria, GlpQ usually degrades the WTA chain by sequentially cleaving the GroP entity from the free end to the PNG-linked end of the GroP-WTA polymer [12]. GlpQ cannot completely degrade glycosylated WTA chains, but can completely degrade unmodified WTA chains. WTA produced by *S. aureus* N315 and SJTU21564 belongs to the RboP type [20,46]. Irrespective of whether the *S. aureus* WTA type is RboP or GroP, its highly conserved linkage in different bacteria species contains two GroP units [8]. This data combined with the results of LBA on *S. aureus* WTA content indicate that the WTA content of *S. aureus* is decreased upon exposure to LBA. Hence, we speculate that GlpQ may degrade RboP-WTA in *S. aureus* by cleaving the phosphodiester bond connecting with 2 GroP units, suggesting that *glpQ* activity is induced in *S. aureus* to function in WTA after inducement with LBA, resulting in a decrease in WTA content and reduced biofilm production.

## 4. Conclusions

*S. aureus* adhesion and biofilm formation can be reduced by LBA. The surface structure of *S. aureus* cells can be disrupted by LBA. Induced production of GlpQ promotes the degradation of WTA in the presence of LBA, which results in a decrease of WTA content that affects the early adhesion stage of *S. aureus* biofilm formation. The findings support the view that WTA hydrolysis is promoted by LBA through the regulation of GlpQ to inhibiting the biofilm formation of *S. aureus*.

## Figures and Tables

**Table 1:** List of primers used for RT-qPCR analysis of the selected genes.

Gene	Primer Sequence (5′-3′)
*glpQ*	F: ATTTATGGCTGCTTCTGCTGT R: GCTTGAAACGTATGCTCTGGT
*sdrC*	F: TGATAAAGATGCCGATGGTGG R: CGCTGTCTGAATCGCTGTCTG
*saK*	F: TGTAGTCCCAGGTTTAATAGG R: CGCGAGTTATTTTGAACC
*clfB*	F: ATAGGCAATCATCAAGCA R: TGTATCATTAGCCGTTGTAT
*rpoB* (housekeeping gene)	F: ATGACTTAGCAAGCGTGGGT R: GCGTTCGATTCAAGTACATCC

## Data Availability

The data of this study are available from the authors upon reasonable request.
